# Enhancing the Prospective Acceptability of a Paediatric Speech and Language Therapy Intervention With Parents From Under‐Represented Communities

**DOI:** 10.1111/hex.70855

**Published:** 2026-08-30

**Authors:** Lucy Rodgers, Nicola Botting, Ros Herman

**Affiliations:** ^1^ City St George's, School of Health and Medical Sciences London UK; ^2^ Sussex Community NHS Foundation Trust, Brighton General Hospital Brighton UK

**Keywords:** intervention development, PPI, speech and language therapy

## Abstract

**Background:**

Integrating the perspectives of people with lived experience is integral to developing new healthcare interventions. However, individuals from under‐represented communities may face additional barriers to taking part in such patient and public involvement (PPI) activities. Within paediatric speech and language therapy (SLT), under‐represented communities might include families from lower SES backgrounds and/or families where the majority language for that country is not spoken. Excluding families from under‐represented communities poses the risk of new interventions not being acceptable to them, thus exacerbating pre‐existing healthcare inequalities.

**Objective:**

The aim of this study was to optimise the prospective acceptability of a new paediatric speech and language therapy intervention with parents from under‐represented backgrounds, in the hope of optimising future accessibility to families from diverse communities.

**Methods:**

An individualised PPI approach was taken, consisting of two semi structured interviews informed by the Theoretical Framework of Acceptability (TFA). Six parent partners from non‐majority language and/or lower SES backgrounds were recruited through a local charity. In the first interview, parent partners were shown a video of the intervention and identified key areas for increasing acceptability. Refinements were made to the intervention by the project steering group in an online meeting. These refinements were the topic of the second interview, where the impact on prospective acceptability was explored.

**Results:**

Following the first round of interviews, areas for improvement included consideration of access to/co‐ordination with wider services, and the financial and practical implications for parents accessing the intervention. Most of the intervention refinements and solutions to potential barriers came directly from the parent partners themselves, rather than the steering group. In the second round of interviews, parent partners rated the intervention's prospective acceptability between 4/5 (acceptable) and 5/5 (very acceptable).

**Conclusion:**

By working with parent partners from under‐represented communities, the research team was able to enhance the prospective acceptability of a new paediatric speech and language therapy intervention. Many refinements related to the wider healthcare and support system, rather than to the content of the intervention itself. This reinforces the importance of exploring and addressing wider contextual barriers within the intervention development process.

**Patient or Public Contribution:**

The methods for this project were co‐designed with a project steering group consisting of 3 Speech and Language Therapists (one with specialist EDI expertise), a bi/multilingual education support worker, a specialist early years teacher, a parent of a child with developmental language disorder (DLD) and an adult with DLD. The steering group also assisted with identifying potential intervention refinements. The parent partners were responsible for identifying areas where acceptability could be increased and addressing the prospective acceptability of the intervention refinements.

## 1 Introduction

Patient and public involvement (PPI) is integral to the development of new healthcare interventions and can enhance future intervention relevance and applicability [1]. However, public partners from under‐represented communities face additional barriers to participating in PPI, which may serve to (unintentionally) reinforce pre‐existing healthcare inequalities [2]. We are developing a novel complex intervention for pre‐school children with co‐occurring features of a speech sound disorder (SSD) and Developmental Language Disorder (DLD), called SWanS (Supporting Words and Sounds; [3, 4]. SWanS involves direct work between the Speech and Language Therapist (SLT) and child in clinic, as well as the SLT supporting the child's parent/s to carry out aspects of the intervention at home. Although *any* child may present with co‐occurring SSD/DLD features, it is known that children from lower socio‐economic status (SES) backgrounds are disproportionately impacted by communication needs in early childhood [5]. Additionally, the voices of families from non‐majority language speaking backgrounds are not routinely incorporated into the development of new paediatric speech and language therapy interventions [6], despite known impacts on therapy accessibility [7]. Therefore, this PPI work aimed to enhance the prospective acceptability of SWanS to parents from lower SES and/or non‐majority language backgrounds, with a view of optimising future accessibility.

### 1.1 Developmental Language Disorder (DLD) and Speech Sound Disorder (SSD)

Features of developmental language disorder (fDLD) are present in approximately 7% of 4‐year‐olds [8]. fDLD may include difficulties in learning and using words, understanding spoken language, and/or putting words together into sentences [9]. fDLDs are closely related to, but separate from, speech sound disorder (SSD). SSD is characterised by difficulties in producing the speech sounds in words, with prevalence at 4 years estimated to be approximately 3.4% [10]. It has been found that as many as 40% of 4‐year‐olds with SSD have fDLD [10]. Despite this high level of co‐occurrence and known negative associations with future literacy [11], mental health [12] and persisting communication needs [13], there are few implementable interventions available to speech and language therapists (SLTs) who work with this clinical group [14]. Therefore, the development of new interventions for children with a co‐occurring SSD/fDLD profile is a priority within the field.

### 1.2 Under‐Represented Groups in Paediatric Speech and Language Therapy

Within the United Kingdom, paediatric early years’ SLT intervention primarily involves both direct work with an SLT in clinic and therapy activities at home with a supported parent [15]. Delivering therapy with supported parents has been evidenced to effect positive change in children with both SSD and fDLD [16, 17]. In order to achieve this, parental engagement (and the SLT effectively supporting this) within the intervention process is crucial [18, 19]. However, for families from under‐represented backgrounds, additional barriers may prevent them from participating in joint SLT‐parent‐led interventions [20]. Within paediatric SLT, specific barriers may be experienced by parents whose first language is not the majority language for that country (e.g., English in the United Kingdom), and parents from lower socio‐economic status (SES) backgrounds, as discussed below.

### 1.3 Low SES Backgrounds

It is widely known that young children from lower SES backgrounds are disproportionately affected by speech and language needs (including SSD and fDLD), with studies highlighting that as many as 40%–50% of children from disadvantaged areas start school with significantly poorer speech and language skills than expected for their age [5]. Within the paediatric SLT context, general barriers to intervention access for parents may include time pressure and differing attitudes/expectations for the intervention process [20, 21, 22]. However, wider healthcare literature highlights that in addition to general barriers to healthcare access faced by all families, families from low‐income backgrounds face additional challenges [23]. As it is children from lower SES backgrounds who are most in need of support, failing to address such barriers when developing new interventions poses the risk of the intervention not being fit for purpose [24]. Therefore, it is imperative that families from lower SES backgrounds are actively (and meaningfully) involved in the intervention development process, as they are best placed to identify key areas to address based on their lived experience.

### 1.4 Non Majority Language Speakers

Historically, families from non‐majority language backgrounds have also been under‐represented within the development of new speech and language therapy interventions [6]. Paediatric speech and language therapy Interventions for non‐majority language speakers have unique qualities, such as delivery through an interpreter [7], and therefore warrant their own exploration within the intervention development process. We know that bi/multilingualism is *not* a cause of speech and language difficulties, and that home language maintenance is important for cultural identity and family relationships [25]. These positive factors around home language maintenance further emphasise the importance of addressing linguistic diversity within the development of new SLT interventions for all children. To more fully understand how to successfully achieve this, parents with relevant lived experience need to be involved. Unfortunately, people from non‐majority language backgrounds may be less likely to participate in healthcare research, due to factors such as limited awareness of opportunities [26]. Therefore, novel PPI approaches are needed to support involvement.

### 1.5 Conducting Patient and Public Involvement (PPI) With Under‐Represented Groups

Within the development of general healthcare interventions (i.e., interventions for a general clinical population rather than a specific demographic group), a lack of diversity is often stated as a limitation to be addressed in the future, rather than one to be addressed from the outset [27]. Unfortunately, this exclusion only serves to widen pre‐existing health inequalities and is economically wasteful [24]. This is why the National Institute of Health Research NIHR [28], a major funder of UK NHS research, has made reducing inequalities by bringing applied research to under‐served communities an explicit part of their 5‐year strategic plan [28]. To address this need, researchers might look to using creative and flexible PPI approaches when seeking to involve people from under‐represented communities within the intervention development process [26, 29]. By addressing the needs of diverse PPI partners, meaningful involvement may still take place, even if it is at a lower intensity [30]. Therefore, such flexible PPI approaches might be employed when seeking to integrate the views of parents from lower SES and/or non‐majority language backgrounds into the development of new paediatric SLT interventions.

### 1.6 Developing the SWanS Intervention (Supporting Words and Sounds)

SWanS is a novel, theory‐based complex intervention for pre‐school children with co‐occurring SSD/DLD features. The intervention development process has been overseen by a project steering group consisting of people with lived experience (an adult with DLD, a parent of a child with DLD), and relevant professionals (3 SLTs, a specialist teacher, a bilingual education support worker). Further information about the steering group co‐design process can be found in Rodgers et al. [3]. Prior to the PPI work with parent partners presented in this paper, core intervention elements for SWanS were co‐designed with the project steering group and agreed with SLTs in a UK‐wide e‐Delphi [4]. This paper presents the final phase of the intervention development process, on refinements to be made to the core intervention elements based on prospective acceptability to parents from lower SES and/or non‐majority language backgrounds. As a robustly developed and frequently applied framework for conceptualising acceptability in healthcare research, the Theoretical Framework of Acceptability (TFA) has been used to underpin this process [31]. It contains 7 core constructs: *affective attitude*, *burden*, *ethicality*, *intervention/system coherence*, *opportunity costs*, *perceived effectiveness* and *self‐efficacy*.

### 1.7 Aim

To enhance the prospective acceptability of a novel preschool speech and language therapy intervention with parents from lower SES and/or non‐majority language backgrounds, with a focus on delivery and coherence.

## 2 Materials and Methods

Ethics for the sharing of information from steering group activities was granted by City St Georges Language and Communication Science Proportionate Review Committee (ethics no. ETH2223‐1673). Ethics for the sharing of information gathered through the involvement of parent partners was also granted by the City St Georges Language and Communication Science Proportionate Review Committee (ethics no. ETH2425‐1402).

A PPI approach was taken. Although there are similarities with qualitative methodologies, a PPI approach differs in that it typically involves individuals being actively involved in conducting the research together *with* the researchers, rather than taking part as participants and data being analysed *on* them [32]. Therefore, a PPI (rather than qualitative) approach was selected for the current project so that parent partners could play an active role and hold more weight in the decision‐making process [32]. However, even within PPI there can be varying degrees of involvement and power sharing, from consultation to collective action [33]. In this project, we aimed for collaboration (i.e., working in partnership) in order to foster collective action (i.e., amendments to the SWanS protocol). An iterative and interactive approach was taken to foster this collaborative (rather than consultative) work, as discussed in Section 2.2 below. The ‘Guidance for Reporting Involvement of patients and the Public’ (GRIPP) 2 short form has been used to guide the writing up of the PPI process [34]; Supporting Information S1).

In an online meeting, the project steering group brainstormed how to facilitate the involvement of parent partners from a diverse range of backgrounds (Supporting Information S2). This included specific consideration of parents facing potential barriers relating to language, socioeconomic status, disability, religion and/or culture. Individual interviews were opted for over focus groups. This was to give each parent more freedom in choosing when, where and how (i.e., in person or online) they could take part. This also aided greater flexibility of pacing, with more time for some parents to process information if needed. The steering group recommended two interviews per parent rather than one, as this would enable each parent to discuss the acceptability of refinements made to the intervention following the first round of interviews.

### 2.1 Recruitment

We sought parents of 3–6 year olds with a non‐majority home language (i.e., non‐English) and/or who were from lower SES backgrounds. Initially, the lead researcher made contact with a local charity, “The Trust for Developing Communities,” who support families from lower SES and/or non‐English‐speaking backgrounds by providing advice and opportunities for community participation. After a meeting with the charity lead to discuss this project, a flyer was developed for community workers to share with parents who they felt might be interested in taking part. Community workers then supported interested parents to read the parent partner information sheet and complete the expression of interest and consent form. To support participation and acknowledge time and expertise provided by the parents taking part, they were reimbursed £50 for each interview, as in keeping with the NIHR payment guidance for public involvement [35].

### 2.2 Structure

**Figure 1:**
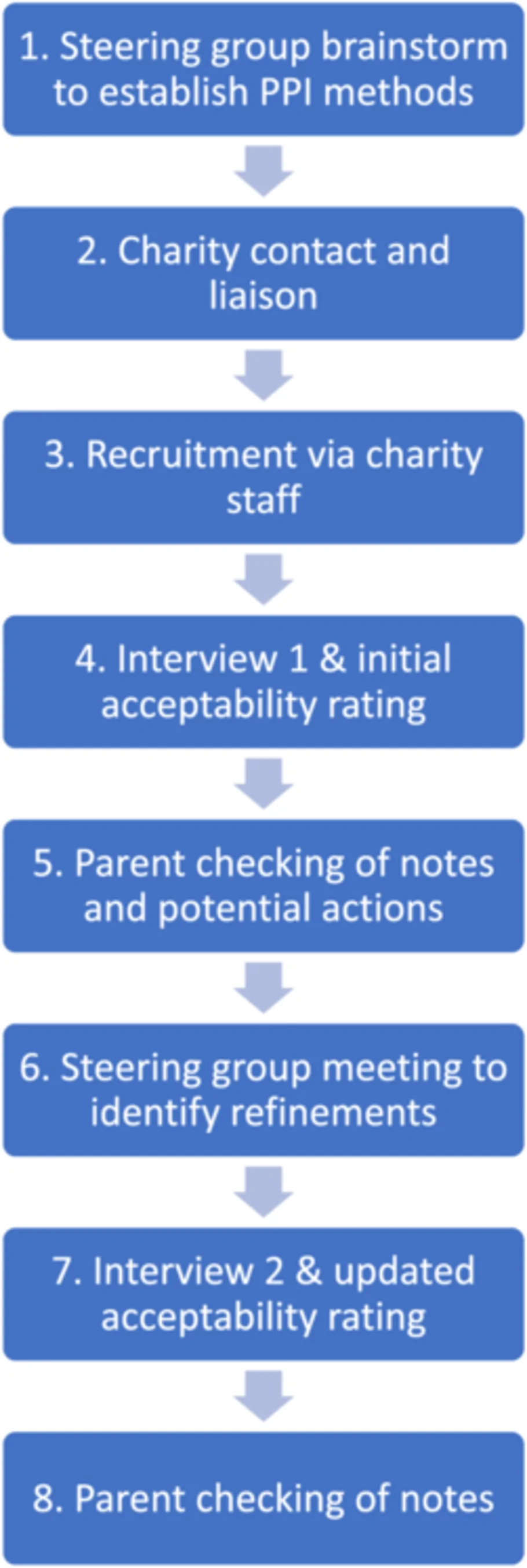
Structure of involvement.

As in keeping with the PPI approach taken, interview data were not analysed using a qualitative methodology. Instead, key information to take forward from the interviews was decided together *with* each parent, as specified below. The structure of this PPI work is also represented visually in Figure 1.

*1. Interview one:* In the first interview, parent partners were asked to watch a video describing the intervention and asked questions based on the TFA [31]. The schedule for interview 1 can be found in Supporting Information S3. The parent partners were also asked to give the intervention a preliminary acceptability rating out of 5. At the end of the interview, each parent partner identified key acceptability points/areas for refinement for the researcher to address with the project steering group. The researcher e‐mailed each parent partner a summary of their conversation for member checking after the interviews.

*2. Steering group meeting:* The steering group members were sent a copy of the parent partner discussion summaries. In a subsequent whole group meeting, steering group members split into two subgroups to discuss potential actions; the actions were recorded on an online interactive whiteboard.

*3. Interview two:* Refinements to the intervention were shared with each parent partner. Parent partners were given the opportunity to provide feedback and make further suggestions. They were asked to re‐rate the prospective acceptability of the intervention out of 5 and complete a short evaluation form about their experience of being involved. Parent partners were given the option of completing the evaluation form online or on paper. For those opting to complete it on paper, the researcher left the room whilst the parent partner completed it and then put it directly into a designated bag with the other evaluation forms.

### 2.3 Development of Materials

Two videos were created, which described the intervention being carried out with hypothetical families. The first video described a family from a lower SES background, the second video described a family from a non‐majority language background. A script for both videos (Supporting Information S4) was developed by the first author, based on the core intervention elements identified in phase 3 of the intervention development project [4]. The scripts were revised and finalised with the project steering group. The steering group members then watched the videos with the corresponding voiceovers and provided feedback on clarity. As a result of their input, some additional images were added to the videos to aid parent partners in their processing of the verbal narrative.

The brief short evaluation form was informed by questions/comments from a steering group brainstorming activity (Supporting Information S5).

## 3 Results

Six parent partners took part; three were from lower SES backgrounds, one was from a non‐ majority language background and two were from both lower SES and non‐majority language backgrounds. The interviews took place online (3 parents) or with the researcher visiting them at home (3 parents), at varied times and days of the week according to their preferences.

### 3.1 Interviews: Round One

When rating the initial overall acceptability of the intervention, parent responses fell between 4 (acceptable) and 5 (very acceptable) (4,4,4,4,4.5,5). However, areas for improving prospective acceptability were identified by all parent partners. These areas related to factors such as wider parental support, minimising parental burden, reassurance around home language use and empowering the parent accessing SWanS with knowledge and understanding. Further details regarding the agreed consideration points and potential refinements suggested by the parent partners are given in Table 1.

**Table 1:** Agreed consideration points/potential refinements following the first round of interviews.

Parent partner	Consideration points/potential refinements
Charlie	Provide a bitesize rationale within the parent/SLT discussion. Include the science behind the strategies. Worries about the future—provide the parent with information at the end of the intervention; not just about next steps but also how to monitor progress, advocate for their child's needs and questions to ask nursery about progress. How can we support the child if their parent becomes upset during a discussion?
Etta	Reminders/check‐ins for parent during the week, e.g., texts. Wider parent support ‐ could be within wider community or charities. Testimonials from parents who previously completed the intervention with advice for parents currently going through it. Financial impact of getting to the therapy session, signpost parents to financial support they are entitled to.
Sarah	Note‐taking at home could be daunting, could structure it into brief bullet points. Signpost to financial/practical support available for getting to the sessions. Be explicit in the intervention description re: reward for putting in the effort. Try to replace the term “therapy” with “support” or “guidance” where possible.
Habiba^a^	The parent may need encouragement if they are worried that they should be using English (if the child is using English at nursery). Some guidance at the start about changes the parent might see and how long it can take—should be honest and realistic as every child is different. After the final session, there should be clear guidance for what the parent can continue doing.
Eszter^a^	Put in a little more information about what to expect (potential changes in the child) at the start and reassurance if not meeting goals. Regarding target 3 (comprehensibility), include advice about how to respond if the child is upset if they are not understood (not just the strategy to make themselves understood, but also validating how they are feeling).
Yemaya^a^	Bringing therapy into the home—when talking about home schedules, also discuss practical aspects such as spaces to do activities. Discuss how the home is physically set up so SLT can get a sense of what it looks like. Record therapy activities to be done at home—this would be a reminder for the parent, which they can also share with other family members. Repetition of key points about bi/multi‐lingualism—weave into whole duration of the intervention (not just one‐off mention at start). The interpreter could be asked to write down key takeaway points in the parents’ home language.

^a^ Non majority language background.

### 3.2 Interim Steering Group Meeting

Summary notes (Supporting Information S6) from the first round of interviews were shared with the project steering group ahead of the online meeting. Potential action points were grouped together into 12 areas for discussion. Table 2 below provides details of the resulting proposed refinements which were discussed with parent partners in the second round of interviews and whether they came directly from parent interviews (PI) or were based on project steering group discussion (SG). For some items, more than one refinement was given. For example, item 3, where if the parent is upset, they have the option to talk to the SLT over the phone before or after the session and/or to bring a supportive person along with them to the session. These options were included so that the refinements could be applied to as many different contexts as possible. In the example of item 3, this was so that parents would have alternative options should a person not be available to accompany them to sessions.

**Table 2:** Proposed refinements following the steering group meeting/round 1 interviews.

Item no.	Item description *(TFA construct/s)*	Refinements
1	It would be helpful for the parent to know *why* the recommended activities would work *(perceived effectiveness and coherence)*	1a. Each activity/technique to have a corresponding rationale from the literature. Put in plain English. (PI)
2	What happens after the final session? Need to consider the long term, including the parent advocating for their child's needs and continuing supporting them at home *(self‐efficacy)*	2a. Final session—give the parent tailored information on what to look out for, supporting progression, monitoring progression. (PI)2b. Include information to share with their educational setting if applicable. (SG)
3	What happens if the parent gets upset in the session? The child may overhear them use negative language about them *(ethicality*)	3a. Brief telephone call prior to the appointment. (SG)3b. Opportunity to speak later over the phone. (SG)3c. Distract child with preferred toys in another section of the room whilst having a quiet chat. (SG)3d. Ask for another family member/friend to come along if a sensitive conversation without the child is needed. (SG)
4	There is a lot to do in the intervention, parents are very busy and may forget to do activities between sessions *(burden and self‐efficacy)*	4a. Non‐English speaking parents—interpreter to write down notes during the session which the parent can take away. (PI)4b. Video recording of the session for the parent to take away (as both a reminder and a means of sharing with other family members). (PI/SG)4c. Simple communication, e.g., tick box of completion of home activities. (SG)4d. Send parents home with a checklist. (SG)4e. Double‐check with parents what means of communication is best for them to make sure they receive reminders. (SG)4f. Symbols next to activities on the checklist so the child can actively take part and check off what they have done (also helps parents who are visual learners). (SG)
5	The parent might feel alone and could benefit from a wider support network *(affective attitude and ethicality)*	5a. Link with national charities (PI)5b. Testimonials from parents who previously completed the intervention (PI)5c. Signpost to social media support group, e.g., Facebook groups (SG)5d. Signpost to YouTube videos to hear about the experiences of other parents. (SG)5e. Non‐majority‐language families (if possible)‐, link with parents from a similar background to share experiences. (SG)5f. All therapy‐related information disseminated to all relevant people in the child's life, e.g., nursery. (SG)5j. Consider how to do testimonials—short video testimonials as well as/alongside written accounts. (SG)
6	There are potential financial implications in having to travel to a session *(opportunity costs)*	6a. Signposting via an information sheet of possible support available. (PI)6b. Signpost to charities or social work team for local support. (PI)6c. May be eligible for DLA (signpost). (SG)6d. If social workers are involved, they may be able to drive families to appointments. (SG)6e. Try to schedule the intervention for the closest possible community setting for the child/one they could potentially walk to. (SG)
7	Note taking at home could be overwhelming *(burden and self‐efficacy)*	7a. Phrase as “brief” note taking. (SG)7b. Provide simple bullet point structure. (SG)
8	There may be negative connotations of the term “therapy” for some parents *(affective attitude)*	8a. Within the intervention description, replace “therapy” with “support” or “guidance” where possible. (PI)8b. Explain what therapy means in the *speech and language therapy* context at the start of the intervention. (SG)
9	There should be clarity about the potential change (reward) for the parent putting in the effort. This should be realistic so the parent does not feel like they are failing *(coherence and self‐efficacy)*	9a. Initial discussion—outline how long it may take but likely positive consequences (reward). (PI)9b. Reassure if slow progress, focus on small gains. (SG)
10	The parent might worry about the child using English in nursery, and it being counter‐intuitive for them to continue with their home language at home *(perceived effectiveness and coherence)*	10a. Repetition of key points (i.e., benefits of home language) throughout the intervention, not just at the start. (PI)
11	The parent may not know how to respond in the moment if their child is dysregulated and upset at not being understood *(self‐efficacy)*	11a. Target area 3 (comprehensibility)—include advice about how to respond if the child is very upset/dysregulated (e.g., validating how they are feeling). (PI)
12	It is important to consider the families’ physical space at home when thinking about how activities will translate to the home context *(perceived effectiveness)*	12a. Initial discussion—cover practical aspects such as spaces to do activities, describing how the home is physically set up so SLT can get a sense of what it looks like. (PI)

*Note:* SG—refinement based on project steering group discussion; PI—refinement came directly from parent interviews.

Although each of the TFA constructs is represented in the intervention refinements, the construct of self‐efficacy frequently occurs due to the parents’ emphasis on how parents might *feel* when taking part in SWanS. For example, their confidence in supporting their child after SWanS has finished, and potentially feeling like they have failed if their child does not make progress.

### 3.3 Interviews: Round Two

The proposed refinements were discussed with each parent partner in the second interview (schedule in Supporting Information S7). When asked to re‐rate the overall prospective acceptability of the intervention following these refinements, ratings ranged from 4.5 (acceptable/very acceptable) to 5 (very acceptable) (5,4.5,5,4.5,5,5). Full discussion summaries (member checked with each parent partner following the second interview) can be found in Supporting Information S8.

Parent partners reported that although the refinements are seemingly small, they could make a big difference to the parent's ability to access the intervention:

I mean, all of them seemed really, um, good development of what I thought was already a positive intervention.

(Etta)

…. little things that are going to help the parents. Just that little bit more.

(Charlie)

Parent partners also responded positively to the refinements suggested by the other parents and the project steering group:

They can hear from other parent's view. And then having the tips or kind of, what to expect. I think that's also it's quite reassuring in a way

(Eszter, when discussing item 5)

And you might not notice the small things and as a parent, it's like the hard moments will stick in your brain longer than the good moments.

(Sarah, when discussing item 9)

During the discussions, parent partners built on the refinements given by providing some further suggestions:

What about if they explain this in a letter, before the appointment. Like some typical information to tell the parent so they know what to expect.

(Habiba, when discussing item 3)

Are there standardised things that you could maybe video and have a link to a YouTube video?.

(Sarah, when discussing item 4)

### 3.4 Evaluation

Directly following the second interview, three parent partners opted to complete the evaluation form online (Charlie, Etta, Yamaya), with the remaining three opting to complete it on paper (Sarah, Habiba, Eszter). As Habiba's interview was with an interpreter, Habiba had the opportunity to request clarification on the evaluation questions before the researcher left the room. A summary of the responses where ratings were given is provided in Table 3 below, with all responses to the open‐ended questions included in Supporting Information S9.

**Table 3:** Evaluation ratings.

Question	Raw ratings from the 6 parent partners	Mean
1. Did you feel like you could be honest with your responses? *Scale from 1 = not at all, to 5 = all of the time*	5,5,5,4,5,5	4.8
2. Did you feel like your responses were valued? *Scale from 1 = not at all, to 5 = all of the time*	5,5,5,4,5,5	4.8
3. How confident would you feel about taking part in similar projects in the future? *Scale from 1= not at all confident, to 5 = very confident*	5,5,5,4,5,5	4.8

Open text comments indicated that the process could have been improved, for example, by having an in‐person interpreter instead of online:

I would have an in person translator.

(Habiba)

Parent partners reported that taking part had been a positive experience, and that they enjoyed contributing to the project and hearing about the solutions posed by the steering group/other parents:

I liked that there was a follow up to hear what other parents thought, to learn how the steering group discussed our concerns/suggestions, and to see how they were acted upon. It felt nice.

(Charlie)

I found the whole thing really interesting, but I particularly enjoyed the follow up session where I could hear how my thoughts had impacted the intervention and other people's ideas that I hadn't considered.

(Etta)

I enjoyed getting new ideas and sharing my information. I can share my experiences and help.

(Habiba)

(I enjoyed) being able to participate and help.

(Eszter)

Aside from the areas of improvement stated above (i.e., having an in‐person interpreter), parent partners were positive about the approach to involvement, particularly regarding flexibility and clarity:

It was a very straightforward and enjoyable process. I didn't feel rushed in any way and it also didn't feel like it was dragging on.

(Charlie)

I appreciated Lucy being flexible and working around my availability.

(Yemaya)

It was well thought out, organised and respectful.

(Eszter)

All parent partners opted in to being contacted by the research team when opportunities to be involved in the project arise in the future.

## 4 Discussion

The aim of this work has been to enhance the prospective acceptability of a novel intervention for pre‐school children with co‐occurring features of SSD/fDLD with parent partners from under‐represented backgrounds. By adopting an iterative semi‐ structured PPI interview approach, we have been able to refine the intervention description to enhance prospective acceptability. Areas for refinement highlighted as important by parent partners include integrating environmental supports (e.g., signposting to other services), empowering parents with knowledge and skills and minimising parental burden where possible. Although the refinements do not impact the core content of the SWanS intervention, parent partners reported that they would enhance accessibility for parents from under‐represented communities. Implications for the intervention, as well as the wider field, are discussed below.

Several proposed refinements related to potential feelings of isolation which a parent may experience when accessing the intervention. Parent partners emphasised the importance of connecting with other parents and/or charity groups. This is in keeping with recent wider research within paediatric SLT SSD and/or fDLD intervention, where parents reported that hearing the experience of others could help them to understand what may lie ahead [20, 36]. Although *any* parent accessing SWanS may experience isolation, this may be even more so for parents from under‐represented communities [37]. This has implications for the operationalisation of the refinements addressing isolation (e.g., testimonials from other parents); if these refinements are not operationalised with inclusivity in mind, they pose the risk of further marginalisation of already under‐represented groups [37]. Such risks might be mitigated by parent partners from under‐represented communities leading on the development of intervention resources and ensuring that the testimonials reflect a diverse range of cultural and social‐economic backgrounds.

The minimising of parental burden was a key factor relating to acceptability for parent partners. This aligns with the wider field of pre‐school SSD and/or fDLD intervention, which has evidenced that parents require personally tailored support to carry out activities at home [20, 36]. However, it is important to consider that parents from under‐represented groups are more likely to face burdens over and above those of parents from non‐minoritised groups [2, 37]. This was reflected in parent partner comments, such as the cost of getting to a therapy session and external pressures to use English (instead of a preferred home language). This poses a challenge to intervention developers, as many of these wider problems require large‐scale, systematic solutions [38]. Developing such solutions is beyond the scope of a speech and language therapy intervention. However, recent attention has been drawn to the value of integrating public health concepts into paediatric speech and language therapy interventions by broadening actions within the interventions themselves [39]. This “broadening” was organically identified by the parent partners, who suggested wider public health‐related refinements such as the signposting of parents to local charities and other relevant services. Although SWanS may not have the power to impose wider systematic change, this shows the potential value of integrating smaller actions to increase acceptability among parents from under‐represented backgrounds.

### 4.1 Enabling Participation by Using a Personalised PPI Approach

The adoption of a flexible PPI approach centred on when, where and how the individual wants to take part has enabled parent partners from under‐represented communities to take part. This was indicated by parent partner's comments on the evaluation form, no parent partners dropping out, and all parent partners wanting to be contacted about future opportunities to be involved. Working with a local charity to recruit parents had multiple benefits. It meant that parents were able to discuss the project with support from individuals they already had positive relationships with, which likely helped ease potential anxieties and enable them to engage with the unfamiliar researcher [30]. Additionally, all parents had benefited from prior support from the charity, which may likely have been a potential motivator for wanting to take part. Such altruistic rationales for taking part in PPI and wanting to “give back” are not unusual [40].

Our success with this personalised PPI approach supports an emerging shift towards multidimensional and multi‐layered PPI approaches, which acknowledge the complexity of lived experience and barriers to taking part in research [26, 41]. The PPI conducted here moved from “consultation” to “collaboration” [30] as, by having two interviews (and interim sense checking), the research team were able to have a *continuing* conversation with each parent partner and ensure that refinements were meaningfully integrated into the intervention. However, it is vital to have an equity focus throughout the intervention development‐evaluation process [42]. For SWanS, future PPI work can build on the relationships established between each parent partner and the research team with a view to moving towards “public led” involvement [30]. For example, supporting parent partners to lead on the creation of intervention resources would further minimise the power differentiation between parent partners and the research team going forward [43].

### 4.2 Considerations for Wider Research and Clinical Practice

Our project has demonstrated how a highly flexible and individualised PPI approach can facilitate the meaningful input of people from under‐represented communities in healthcare research. However, we might also consider the potential value of other approaches which were not taken. Within their feedback, some parents reported that they greatly valued seeing other parents’ comments; had this work been conducted as a group co‐design workshop instead, parents would have had the opportunity to directly converse with each other and potentially build on each other's ideas. However, the trade‐off would be less flexibility for the parents to have a tailored involvement experience (i.e., in person or online, at a time that suits them, with ample time and space for an interpreter to facilitate conversation). Based on the parents’ feedback, in the future researchers might consider how best to facilitate the “connection” between disparate PPI contributors in such contexts. For this project, the researcher allocated time to feedback to each parent about what each other had reported. In the future, creative methods might be utilised to personalise this strategy further; for example, showing the parent short video clips of the other parents summarising their thoughts.

When considering implications for wider clinical practice, some caution is warranted due to the specific focus on SWanS as well as a relatively small number of parents being involved. The research team cannot rule out that additional information may have been gleaned from involving a larger number of parents. Furthermore, families from lower SES and non‐majority language backgrounds are by no means mutually exclusive groups. This project found that although there were similarities between these groups (e.g., a shared need for signposting to sources of support), some considerations were specific to one or the other (e.g., having an interpreter present). These complex overlaps and differences, as well as the importance of taking an intersectional approach, have also been discussed in recent literature [44]. However, SWanS does share characteristics with other pre‐school SLT interventions, particularly in regard to parent delivery, and so tentative wider learnings may still be gleaned. For example, all parent partners highlighted the importance of a holistic approach, where public health principles may be infused into pre‐existing interventions and care pathways. Constraints on clinical time and resources could make such additional work unfeasible for SLTs, particularly if they work within a universal healthcare (e.g., NHS) context [45]. A straightforward but impactful way to address this would be to create a resource sheet of local support networks, services and charities, for the SLT to give to the parent when they are first seen. This would be a simple way of ensuring that the family is aware of the other sources of support available to them. As indicated by the parent partners in this project, access to such support could make or break a parent being able to engage in the therapy process.

## 5 Limitations

Although our flexible PPI approach was largely successful, it was not perfect. For example, one parent partner fed back that they would have preferred an in‐person interpreter. Interestingly, this parent partner also gave slightly lower ratings (four instead of five) on the evaluation form regarding how good the experience of being involved was for them. This indicates that although their overall experience was positive, having an in‐person interpreter may have enabled them to enjoy being involved as much as the other parent partners. Unfortunately, there were very few interpreters nationwide available to interpret in person for this parent partner's language, with implications for the project timescale. Based on our experience, we would encourage researchers to consider which languages they are likely to need for in‐person interpretation when submitting funding proposals. This would enable them to allocate additional funds for languages with few in‐person interpreters, and incorporate the time needed to find suitable interpreters into their project methodologies.

It is important to note that prospective acceptability differs from retrospective acceptability (i.e., when the acceptability of the intervention is explored *after* the parent partner has experienced the intervention for themselves) [31]. High prospective acceptability does not guarantee acceptability within practice. Additionally, although the parent partners were from under‐represented communities, parents from under‐represented communities with children with speech/language needs might have additional insights. A decision was made to recruit under‐represented parents (directly from a charity) with lived experience of raising young children because they would be well placed to imagine what it would be like for parents in a similar context to them attending an SLT intervention for the first time. However, this is not the same as parents actually *experiencing* the intervention and supporting their child with co‐occurring SSD/DLD features. The intervention development‐evaluation process is cyclical [1], and it will be important to continue involving parents from lower SES and non‐majority language communities when they have experienced the intervention in the trialling process [43]. This continued inclusion would enable a more in‐depth appraisal of the acceptability of the SWanS intervention content, whilst it is being experienced. Additionally, this future work should include a larger number of parents from under‐represented backgrounds, to facilitate an even more diverse representation. One benefit of exploring prospective acceptability is that it provides some focus for future acceptability work (i.e., being able to review the impact of the specific refinements identified).

A final potential limitation of this project is that children themselves were not involved with establishing the prospective acceptability of SWanS. Addressing this will be particularly important in the next stage of the intervention development‐evaluation process, with the researchers aiming to gain feedback on the child's experiences of taking part and to collaborate with them to make the intervention as fun and child‐friendly as it can be. Creative methods such as drawing could be used to achieve this [46].

## 6 Conclusion

The prospective acceptability of a novel intervention for pre‐school children with co‐occurring features of SSD/fDLD has been enhanced by applying a flexible, responsive PPI approach with parent partners from under‐represented backgrounds. The refinements serve as a reminder that healthcare interventions exist within a wider ecosystem of healthcare and social policy, and that wider strategies for health promotion may be just as important for intervention acceptability as the intervention content/delivery mode itself. The impact of the intervention refinements identified will be reviewed within retrospective acceptability work as a part of subsequent trialling.

## Author Contributions

**Lucy Rodgers:** conceptualisation, methodology, writing – original draft, writing – review and editing, resources, project administration, formal analysis, investigation, funding acquisition, visualisation, validation, software, data curation. **Nicola Botting:** supervision, writing – review and editing, writing – original draft. **Ros Herman:** supervision, writing – review and editing, writing – original draft.

## Ethics Statement

Ethics for the sharing of information from steering group activities was granted by the City St Georges Language and Communication Science Proportionate Review Committee (ethics no. ETH2223‐1673). Ethics for the sharing of information gathered through the involvement of parent partners was granted by the City St Georges Language and Communication Science Proportionate Review Committee (ethics no. ETH2425‐1402).

## Conflicts of Interest

The authors declare no conflicts of interest.

## Supporting information


Supporting File 1

Supporting File 2

Supporting File 3

Supporting File 4

Supporting File 5

Supporting File 6

Supporting File 7

Supporting File 8

Supporting File 9

## Data Availability

Data are available in the supporting information of this article.
