# Supplementary material for: Enhancing the Prospective Acceptability of a Paediatric Speech and Language Therapy Intervention With Parents From Under‐Represented Communities
Source: Health Expect. 2026 Aug 30;29(5):e70855. doi: 10.1111/hex.70855 (PMC13527411; doi:10.1111/hex.70855)
Supplement: Supplementary file 1 [file HEX-29-e70855-s001.docx]

**Supplementary material 6**

**Summary notes (interview one)**

Please note: names given (Sarah, Kai, Anita, Ahmed) relate to the characters from the videos shown to each parent partner

**Discussion 1a. Summary**

1.Generally, seems “doable’- not overwhelming or highly technical, building on current knowledge and skills

2.“Ripple effect” of bonding, activities the parent (Sarah) enjoys too

3.“Lots on Sarah”, what about wider support networks?

4.Journey for Sarah to even attend the first session- sense of achievement already, may face pressure, worried about failing

5.Ending of sessions, journey doesn’t ever really stop for Sarah. What can she look out for in the future? What can she share with nursery/future school? Questions to ask them. Future milestones and advocating for needs.

6.Helpful to know scientific rationale behind each activity (in plain language). Deliver in a bitesize way. Need to be careful not to overwhelm her.

7.Kai (child) will be there when Sarah is talking to the SLT. What if it is negative and/or highly emotional? Sarah needs to have space to be honest.

8.How SLT speaks to Sarah is important. Clinic could be intimidating. Parent might feel like they are being graded.

9.Good to be flexible with activities and work within Sarah’s own “parenting rhythm”.

**Rating:** Acceptable 4

Potential action points:

1. Provide bitesize rationale within parent discussion, science behind the strategies
2. Worries about the future- provide information at the end of the intervention not just about next steps but what to look out for and advocating for needs/ questions to ask nursery about progress
3. How can we support the child if their parent becomes upset during a discussion? (could impact child’s confidence)

**Discussion 2a. Summary**

1.Highly collaborative and positive, “clearly thought out”, helpful to have consistent session structure.

2. Goal orientation- clear what they are working towards, what is expected each week, and how it might be measured/what it looks like.

3.A lot of effort for Sarah, particularly in between sessions. Engagement more difficult then (particularly if she is struggling). What would remind her (texts?)/check ins

4.If Sarah is struggling it’s good that she has regular opportunities to discuss and work through in each session

5. Important that Sarah being shown how to do it first

6.Main financial impact-getting to the therapy session

7. Potential positive financial impact- free things to do at home, otherwise may buy lots of toys from anxious googling

8. Feeling of relief to have SLT to work things through with

9.What about wider peer support? Link with other parents doing the intervention if possible

10.How much of the intervention will be tailored (activities themselves)? How? Important to meet needs of individual families.

**Rating:** Acceptable 4

Potential action points:

1. Reminders/ check-ins for parent during the week e.g. texts (automate?)
2. Wider parent support. Could be within wider community (e.g. signpost to Naplic or Afasic). Testimonials from parents who previously completed the intervention with advice for parents currently going through it?
3. Financial impact getting to the therapy session, signpost parents to financial support they are entitled to

**Discussion 3a. Summary**

1.Clear layout of sessions, seems manageable- helps that it’s built around developing skills in day to day life with the child

2. Note taking could be overwhelming (picture shows a long paragraph), can’t wite an essay, would be better as bullet points and framing as “brief notes”

3.Logistics and money- may live miles away, bus fare, parking charges- print out or signpost to support available?

4.”no cost to being at home”

5. Parent will need to come in with a certain level of commitment

6. Would help to be explicit about the reward the parent is getting for putting all the hard work in- e.g. understood at home

7. Helpful to have the clear goals

8. Term “therapy” used a lot, could be off putting to families who have negative connotations of the term (particularly at the start)- use terms support/guidance

9.Window animation off-putting

**Rating:** Acceptable 4.5

Potential action points:

1. Note taking at home, structure it so brief bullet points
2. Signpost to financial/practical support available for getting to the sessions
3. Be explicit in the intervention description re: reward for putting in the effort
4. Try to replace the term “therapy” with “support” or “guidance” where possible

**Discussion 4a. Summary**

1.”We have to help one another”. Importance of helping the community.

2. Challenge- maintaining home language when English is spoken in Nursery. Ahmed might start using English at home instead.

3.It’s good that Anita is getting lots of support to do things at home.

4. It’s clear that Anita could help Ahmed. If she couldn’t help him, the therapist is there to support her.

5. Anita may feel “a little afraid” at the start. She may wonder how long it will take until Ahmed improves. Top of FormBottom of Form

6. It’s important to have an interpreter if Anita doesn’t have enough vocabulary in English.

7. Anita will probably want to continue with activities at home after the sessions have ended. It’s important it is clear what these are.

**Rating:** Acceptable (4)

Potential action points:

1.Encouragement for parent if they are worried that they should be using English if child is using it at nursery

2.Some guidance at start about changes the parent might see and how long it can take- be honest and realistic as every child is different

3.After final session, clear guidance for what the parent can continue doing

**Discussion 5a. Summary**

1.Very good to ask the child what they enjoy and involve them in the decision making- they have a problem with speech, and the adults around them are getting them to be heard

2. Wonder how it would work for different sounds within the Urdu, they might be different to English

3.Definately more of a burden for Anita, she will have to pay more attention at home, but she should prioritise supporting Ahmed.

4. It’s good that the SLT is checking in with Anita frequently and that she is given informative written guidance to take away

5. Anita will need reassurance, but she can be given this as they look back at the goals and progress Ahmed has made together

6. Good to have clear goals to work towards

7. Anita’s confidence might be impacted if Ahmed is making limited progress towards the goals- would be good to have some expectation as to what she might expect and reassurance if little progress

8. How ‘doable’ it is at home could potentially be impacted by how many times a day Anita is expected to do the activities and for how long- every day for 10-15 minutes should be manageable

9.Important to be given a lot of knowledge at the beginning about what to expect- would help with confidence

10.Would also be helpful to have support on how to manage the child’s emotions/how to respond when you don’t understand them and they are upset

**Rating:** Very acceptable 5

Potential action points:

1. Put in a little more information about what to expect (potential changes in the child) at the start and reassurance if not meeting goals
2. Regarding target 3 (comprehensibility), include advice about how to respond if the child is upset if they are not understood (not just the strategy to make themselves understood, but also validating how they are feeling)

**Discussion 6a. Summary**

1. Quite nerve wracking when you don’t know what to expect but “sounds nice and collaborative”

2. 3 areas to work on with focus on impact “making a difference” + “more meaningful”

3. “Quite involved” with extra work for Anita to do, working around clubs, family life etc- good that that the increase in responsibility is gradual across the therapy sessions

4. Good that it’s building on Ahmed’s interests and is focused on gradually building Anita’s skills

5. “It’s helping to really embed it”, really look at home life and how to bring the therapy home, good space to do the activities in, what their day looks like

6. “Everyone’s starting place is quite different”, confidence should increase gradually, “I understand the feeling of wanting to go to someone-you do it”

7. Possible to record parts of a session to watch back at home? Share with other family members

8. Uncertainty about when the child gets older, what about primary school and beyond- good to have this understanding as a foundation

9.Important that parent is re-assured about bilingualism, needs to be repeated as may be counter-intuitive to the parent re: continuing in another language when the child has difficulties

10.Session’s look quite full and fast paced, scope for interpreter to write down notes for Anita to take away?

**Rating:** Acceptable 4

Potential action points:

1. Bringing therapy into the home- when talking about home schedules, covering practical aspects such as spaces to do activities, describing how the home is physically set up so SLT can get a sense of what it looks like
2. Record therapy activities to be done at home- reminder for parent and can share with other family members
3. Repetition of key points about bi/multi-lingualism- weave into whole duration of the intervention (not just on off mention at start)
4. Interpreter to be asked to write down key take away points within the parents home language
