# Supplementary material for: Enhancing the Prospective Acceptability of a Paediatric Speech and Language Therapy Intervention With Parents From Under‐Represented Communities
Source: Health Expect. 2026 Aug 30;29(5):e70855. doi: 10.1111/hex.70855 (PMC13527411; doi:10.1111/hex.70855)
Supplement: Supplementary file 2 [file HEX-29-e70855-s002.docx]

**Supplementary material 4**

**Video scripts**

**Script 1- Anita and Ahmed (non-majority home language)**

**Scene 1 (introduce characters)**

- This is Ahmed, he is 3 and a half.
- This is Ahmed’s mum, Anita.
- They speak Urdu at home. Anita doesn’t know much English. Ahmed knows a little from nursery.
- Ahmed finds it difficult to say certain sounds.
- He also finds it difficult to use words.
- This means that other people often cannot understand him.
- This makes him upset and impacts his confidence.
- Anita is worried about Ahmed.
- She has referred him to speech and language therapy for help.

**Scene 2 (SLT assessment)**

- Anita takes Ahmed to a speech and language therapy assessment at a local clinic. She brings in some of Ahmed’s toys from home to help make him feel more comfortable.
- Anita and the speech and language therapist talk about what speech and language therapy is and how it can help Ahmed. Including how, as his communication skills develop, his frustration will reduce.
- An Urdu interpreter is there to help, as the speech and language therapist does not speak Urdu.
- Ahmed’s assessment shows that he needs therapy to develop his speech and language.
- The therapist reassures Anita that Ahmed’s difficulties are not because he is bilingual. The therapist also tells Anita that it is important that Anita continues to use Urdu at home.
- The therapy will involve Anita bringing Ahmed for therapy sessions with the therapist.
- The therapist explains that the therapy will also involve Anita doing activities with Ahmed at home in between their sessions. We know from research that doing activities at home is essential to making the therapy effective.

**Scene 3 (first therapy session)**

- Anita and Ahmed are at their first therapy session.
- An Urdu interpreter is there to help again. They will be at all the therapy sessions.
- In the first part of the session, the therapist and Anita discuss Ahmed’s interests and home life in more depth. This will help the therapist to make the therapy activities more relevant and fun for Ahmed.
- They also talk about potential difficulties Anita might have with attending sessions, or doing the activities at home, and what might help her to overcome this.
- They then discuss and agree on 3 targets to focus on. Target one is for Ahmed to use more words. Target two is for Ahmed to better understand how to use the different sounds in words. Target 3 is for Ahmed to be more understandable to others, using strategies in everyday life. This is to help reduce his frustration in the short term, whilst the clarity of his speech is still developing.
- In the second part of the session, the therapist and Anita talk about this third target in more detail. They choose a strategy Anita would like to try that week, to see how it helps. The strategy Anita selects to use when she does not understand what Ahmed is saying is asking him to ‘show her’ what he means, using gesture, pointing or pictures.
- Anita will try out this strategy with Ahmed at home, before the next session.

**Scene 4 (second therapy session)**

- Anita and Ahmed are at the second therapy session.
- They start of by recapping how the “show me” strategy went. The therapist and Anita adapt the strategy or change it to another one, depending on how things went.
- In the second part of the session, Anita watches the therapist do some direct activities with Ahmed, with the support of the interpreter. They are to help with targets one-using more words, and two-understanding how different sounds are used within words. The activities include toys Ahmed finds interesting. Most of the activities involve Ahmed listening to the speech and language therapist, unless he feels comfortable enough to speak.
- In the third part of the session, Anita then has a go at one or two of these activities herself, with the support from the therapist.
- The therapist and Anita then discuss together what went well. They also ask Ahmed what he thought about the activities.
- As a team, they set goals for what Anita could do with Ahmed at home. During this discussion, the therapist focuses on the positives to build Anita’s confidence.
- In the final part of the session the therapist and Anita talk about what might help Anita to do these activities at home, and what might get in the way. They then plan for how Anita will do the activities. This includes how many times Anita might carry them out, and for how long.
- Anita is also given written guidance take away. This will remind her of what they talked about in the session.

**Scene 5 (home practice)**

- Anita does the activities at home with Ahmed, as planned. She also continues with the “show me” strategy they identified in session 1.
- Anita makes notes of what they do during the week and how Ahmed is responding.
- This helps her to keep track of their activities together.

**Scene 6 (third therapy session)**

- In their third therapy session, Anita discusses her notes with therapist. They talk about what went well and what might need to be changed.
- As with the previous therapy session, Anita then watches the therapist do some more activities with Ahmed. She then has a go herself.
- Like the last session, the therapist and Anita discuss together what went well, and also ask Ahmed what he thinks. They set goals for what Anita could do the following week at home and look back at Anita’s notes from the last week when doing this.
- They then set a plan together for what Anita will do at home the following week.

**Scene/slide 7: General outline**

By working as a team, the therapy sessions continue in this way:

- 1.Anita and the therapist discuss Anita’s notes from her home activities with Ahmed
- 2.The therapist does direct activities with Ahmed, with the support of an interpreter
- 3.Anita practices some activities with the therapist’s guidance. Over time, the therapist provides less guidance, so that Anita can become more independent in carrying out activities.
- 4.Anita and the therapist make a joint plan for what Anita can do at home that week, considering what activities Ahmed says that he enjoys.

**Scene 8 (final session)**

- It is the last session with the therapist.
- The therapist and Anita review their targets together.
- They talk about changes they have seen in Ahmed, and any changes other family members may have noticed.
- The therapist recommends to Anita what she can continue doing at home.
- They talk about what will happen next in terms of support from the speech and language therapy service.
- Anita is given a contact number she can call if she has any questions before they access the service again.

**Script 2- Sarah and Kai (low SES)**

**Scene 1 (introduce characters)**

- This is Kai.
- Kai is 3 and a half.
- This is Kai’s mum, Sarah.
- Sarah has noticed that Kai finds it difficult to say certain sounds.
- He also finds it difficult to use words.
- This means that other people often cannot understand him.
- This makes him upset and impacts his confidence.
- Sarah is worried about Kai.
- She has referred him to speech and language therapy for help.

**Scene 2 (SLT assessment)**

- Sarah takes Kai to a speech and language therapy assessment at a local clinic. She brings in some of Kai’s toys from home to help make him feel more comfortable.
- Sarah and the speech and language therapist talk about what speech and language therapy is and how it can help Kai. Including how, as his communication skills develop, his frustration will reduce.
- Kai’s assessment shows that he needs therapy to develop his speech and language.
- The therapy will involve Sarah bringing Kai for therapy sessions with the therapist, in clinic.
- The therapist explains that the therapy will also involve Sarah doing activities with Kai at home in between their sessions. We know from research that doing activities at home is essential to making the therapy effective.

**Scene 3 (first therapy session)**

- Sarah and Kai are at their first therapy session.
- In the first part of the session, the therapist and Sarah discuss Kai’s interests and home life in more depth. This will help the therapist to make the therapy activities more relevant and fun for Kai.
- They also talk about potential difficulties Sarah might have with attending sessions, or doing the activities at home, and what might help her to overcome this. Sarah mentions that things have been a bit difficult recently and that the families rent has gone up.
- They then discuss and agree on 3 targets to focus on. Target one is for Kai to use more words. Target two is for Kai to better understand how to use the different sounds in words. Target 3 is for Kai to be more understandable to others, using strategies in everyday life. This is to help reduce his frustration in the short term, whilst the clarity of his speech is still developing.
- In the second part of the session, the therapist and Sarah talk about this third target in more detail. They choose a strategy Sarah would like to try that week, to see how it helps. The strategy Sarah selects to use when she does not understand what kai is saying is asking him to ‘show her’ what he means, using gesture, pointing or pictures.
- Sarah will try out this strategy with Kai at home, before the next session.

**Scene 4 (second therapy session)**

- Sarah and Kai are at the second therapy session.
- They start of by recapping how the “show me” strategy went. The therapist and Sarah adapt the strategy or change it to another one, depending on how things went.
- In the second part of the session, Sarah watches the therapist do some direct activities with Kai. They are to help with targets one-using more words, and two-understanding how different sounds are used within words. The activities include toys Kai finds interesting. Most of the activities involve Kai listening to the speech and language therapist, unless he feels comfortable enough to speak.
- In the third part of the session, Sarah then has a go at one or two of these activities herself, with the support from the therapist.
- The therapist and Sarah then discuss together what went well. They also ask Kai what he thought about the activities.
- As a team, they set goals for what Sarah could do with Kai at home. During this discussion, the therapist focuses on the positives to build Sarah’s confidence.
- In the final part of the session the therapist and Sarah talk about what might help Sarah to do these activities at home, and what might get in the way. They then plan for how Sarah will do the activities. This includes how many times Sarah might carry them out, and for how long.
- Sarah is also given written guidance take away. This will remind her of what they talked about in the session.

**Scene 5 (home practice)**

- Sarah does the activities at home with Kai, as planned. She also continues with the “show me” strategy they identified in session 1.
- Sarah makes notes of what they do during the week and how Kai is responding.
- This helps her to keep track of their activities together.

**Scene 6 (therapy session)**

- In their third therapy session, Sarah discusses her notes with the therapist. They talk about what went well and what might need to be changed.
- As with the previous therapy session, Sarah then watches the therapist do some more activities with Kai. She then has a go herself.
- Like the last session, the therapist and Sarah discuss together what went well, and also ask Kai what he thinks. They set goals for what Sarah could do the following week at home and look back at Sarah’s notes from the last week when doing this.
- They then set a plan together for what Sarah will do at home the following week.

**Scene/slide 7: General outline**

By working as a team, the therapy sessions continue in this way:

- 1.Sarah and the therapist discuss Sarah’s notes from her home activities with Kai
- 2.The therapist does direct activities with Kai, with the support of an interpreter
- 3.Sarah practices some activities with the therapist’s guidance. Over time, the therapist provides less guidance, so that Sarah can become more independent in carrying out activities.
- 4.Sarah and the therapist make a joint plan for what Sarah can do at home that week, considering what activities Kai says that he enjoys.

**Scene 8 (final session)**

- It is the last session with the therapist.
- The therapist and Sarah review their targets together.
- They talk about changes they have seen in Kai, and any changes other family members may have noticed.
- The therapist recommends to Sarah what she can continue doing at home.
- They talk about what will happen next in terms of support from the speech and language therapy service.
- Sarah is given a contact number she can call if she has any questions before they access the service again
