# Supplementary material for: Enhancing the Prospective Acceptability of a Paediatric Speech and Language Therapy Intervention With Parents From Under‐Represented Communities
Source: Health Expect. 2026 Aug 30;29(5):e70855. doi: 10.1111/hex.70855 (PMC13527411; doi:10.1111/hex.70855)
Supplement: Supplementary file 3 [file HEX-29-e70855-s003.docx]

**Supplementary material 9**

**(Free text) evaluation responses**

**Is there anything you particularly enjoyed about taking part?**

1.I liked that there was a follow up to hear what other parents thought, to learn how the steering group discussed our concerns/suggestions, and to see how they were acted upon. It felt nice.

2.I found the whole thing really interesting, but i particularly enjoyed the follow up session where I could hear how my thoughts had impacted the intervention and other people's ideas that I hadn't considered.

3.Being able to participate and help.

4.I enjoyed getting new ideas and sharing my information. I can share my experiences and help.

5.Knowing **researcher’s name** is trying to make a difference for children and their parents.

6.I enjoyed all of it. It was great having that first chat with **researcher’s name** to hear about the therapy, watch the video and have that initial chat about what it involves.

**Do you have any suggestions for what could have been done differently?**

1.No, it was a very straightforward and enjoyable process. I didn't feel rushed in any way and it also didn't feel like it was dragging on.

2.No I feel it was well put together.

3.I would have an in person translator.

4.It was well thought out, organised and respectful.

5.Not for me. I appreciated **researcher’s name** being flexible and working around my availability.

**3.Please put any other comments you might have here**

3.It has been very enjoyable and I feel it was productive

5.Best of luck!
