# Supplementary material for: Enhancing the Prospective Acceptability of a Paediatric Speech and Language Therapy Intervention With Parents From Under‐Represented Communities
Source: Health Expect. 2026 Aug 30;29(5):e70855. doi: 10.1111/hex.70855 (PMC13527411; doi:10.1111/hex.70855)
Supplement: Supplementary file 4 [file HEX-29-e70855-s004.docx]

**Supplementary material 2**

**Steering group brainstorm (involving parents from diverse backgrounds)**
