# Supplementary material for: Enhancing the Prospective Acceptability of a Paediatric Speech and Language Therapy Intervention With Parents From Under‐Represented Communities
Source: Health Expect. 2026 Aug 30;29(5):e70855. doi: 10.1111/hex.70855 (PMC13527411; doi:10.1111/hex.70855)
Supplement: Supplementary file 7 [file HEX-29-e70855-s007.docx]

**Supplementary material 8**

**Summary notes (interview two)**

**Discussion 1b. Summary**

1.Refinements relating to the 12 key points were very appropriate- *“Each point was really good. Really great.” “All these little things that I think it sounds really good”.*

2.Wider support for multilingual families very helpful + put in contact with local multilingual family hub*-“ I volunteer, as a library and one of the workers at the toy library. She, um, runs the multilingual family's group that's run by the council and the family hub. Some of the family hubs do have multilingual family groups”.*

3.Testimonials positive to have- *“reassurance and positivity going forward”*, not feel so isolated

4.Very helpful to set expectations at the start, reassure parent if little/no progress seen- *“I think that like, as a parent, you really nothing would make you happier than to hear the therapist saying-like, look at them!”*

5.Important for bi/multi-lingual families to be informed of the evidence- *“That's a nice point. And I didn't know the evidence about that. That's really like I can imagine for a family that would be important to hear”*

6. The tweaks to the intervention appear small but could help with access*- “I think it sounds like especially within all those tweaks of like just little things that are going to help the parents. Just that little bit more. And show that, like, you understand their situation”*

7.Good to have video to be able to show partner what happened in the session or what activity/technique was focused on- *“And now you can share your partner as well. Like that. Those are really small things. But actually will make a huge difference for parents”.*

**Rating:** 5- very acceptable

Future things for the researchers to note:

1. Videos of the clinic activities- if confidentiality is an issue, there could be a bank of clips showing the SLT do the activity/technique with a doll
2. Access to multilingual groups may vary by geographical area, will need to find out what is available in the parents’ area.

**Discussion 2b. Summary**

1.Good to be informed of the closest clinic to them geographically but bear in mind this may not be the best still- *“if somebody doesn't, doesn't drive, or is often visiting someone in a different area, it might not be their actually geographically closest children's centre might not be the one they would prefer to visit”.*

2.Very important to have the reassurance and celebration of small wins- “*And then the problem with that is also if there's any measure given, then if that, that could cause extra stress for the parent. If their child isn't measuring up”*

3.Refinements relating to the 12 key points all seemed ok- *“I mean, all of them seemed really, um, good development of what I thought was already a positive intervention”*

4.Interesting to hear about the refinements made in response to the other parents’ points - *“that home space thing as well. I think that that's really, um, I hadn't thought of that. Yeah, could be a massive, uh, influence on whether something works for a family or not. They might feel if it's quite different to what's in the therapy room, they might feel like they are not equipped at home”.*

**Rating:** 4.5- acceptable/very acceptable

Future things for the researchers to note:

1. The closest clinic geographically to the family may not be the easiest for them to get to. Ask first and make the parent aware of options if applicable.

**Discussion 3b. Summary**

1.Good to have clear directions once the therapy block has finished:

*“Where do we go from here. Yeah. Yeah. That's really good”*

2.Good to have alternative forms of communication if discussing matters not appropriate for the child:

*“Or an email address. So like the day before you, they could maybe email their therapist and be like just to let you know Kai's. been really good at this bit, but we're really struggling with this”*

3.Helpful to have examples of activities to view at home:

*“Are there standardised things that you could maybe video and have a link to a YouTube video”*

4.Good to have flexibility of writing bullet points at home or more if the parent wants:

*“And they'll be parents that will write a whole essay and they'll be parents that will just tick the smiley face and be like, yeah, it worked well for us”*

5.Great idea for the therapist to signpost the parent to other potential modes of support:

*“even if it's just an advice sheet”*

*“All on a piece of paper. Stick it to your fridge, please. Um, here's our contact telephone number at the bottom. And email address. Job done. You've got 90% of all your information that you want to give people. All iin one place”*

6.Expaining the context of therapy in speech and language therapy:

*“The whole thing is the guidance and support. We're going to guide you how to support your child and your family. To a goal”*

7.Important for the speech and language therapist to point out the good things/ small improvements:

*“And you might not notice the small things and as a parent, it's like the hard moments will stick in your brain longer than the good moments”*

**Rating:** 5- Very acceptable

Future things for the researchers to note:

1. *“failing that headset music”-* another option for when discussing sensitive conversations
2. “*like a welcome pack or something”.* Consider a parent ‘wellcome pack’ with core information in

**Discussion 4b. Summary**

1.Good to reassure parents that they may not see big changes straight away

“*Because each child is different”*

*“A mother or parent has to be patient”*

2.Important for the parent to follow the guidance given, and this being linked to change seen in the child:

*“the parent has to follow the guidance because some people, they maybe do not follow the steps which they have to”*

3.Parents may feel that there could be a quick fix (like medicine) for their child’s needs, but the child may just need time and the extra support:

*“Some parents may think it is like medicine you can take and get better quickly but they just need time to go with it”*

4.Good to have wider support from other services. Sense of community important.

*“Language therapy can tell them where they can get more wider information to help them”*

*“some of us need it because we have a yearning for a local service place”*

5. Good to ask about preferred location for the therapy if possible. If child has multiple appointments it would be better to have them in one place.

“*We have to take a bus and maybe we'll worry about it as well”*

*“they should refer people to the place where they are because some people, they are referred to different places”*

6. Still important to call it speech and language therapy (use term therapy) because that is what it is:

*“But the word is language therapy. And then you call it just as it is”*

7.Helpful to have acronyms spelt out in full:

*“you use just one letter for whole sentences. And as we are learning English, we find this difficult”.* Important to use *“The full term”*

8. Would be helpful to explain upfront in a letter about options for discussing sensitive issues away from the child:

*“What about if they explain this in a letter, before the appointment. Like some typical information to tell the parent so they know what to expect”*

9.Wider services, would be helpful if health visiting teams could do in person visits:

*“If health visitors also keep visiting like before Covid. Before Covid 19 the health visitor was visiting more regularly”*

10.Good idea for the therapist to ask about the physical space in the child’s home:

*“I think that's a good idea. Some houses can be small, and small for the children”*

**Rating:** 4.5- Acceptable/very acceptable

Future things for the researchers to note:

1. Consider content of letter/intervention overview leaflet for parent, expectations

**Discussion 5b. Summary**

1. *“Actually, really helpful”* for the interpreter to write down notes in the parent’s home language during the session. The parent can then take these home.

2. *“I think that's really helpful”* Good to signpost parents to other sources of support in their local area, as well as giving the parent the knowledge they need:

*“When you first have a child you don't know so many things . And maybe not everyone have the support around them. So mum or whatever, whoever helps. Um, and I remember I had to actually like Google”*

3.Testimonal is a good idea:

*“They can hear from other parent's view. And then having the tips or kind of, what to expect. I think that's also it's quite reassuring in a way”*

4. “*That was interesting point, actually”* about interpretation of the term therapy:

*“And then when you think, oh, maybe physiotherapy, then it's like it has something. Therapy”*

5.Important to point out the positives:

*“Especially when you feel like you didn't really get anywhere. Or maybe you had like a week and everything's gone wrong and the kid didn't really wanted to take part. But then having to see that professional who says like, oh no, look, look, actually you have made that progress. Yeah. I think that's very important”*

**Rating:** 5- Very acceptable

**Discussion 6b. Summary**

1.Good to link in with wider services, and co-ordinating organisations-

*“Even linking in very closely with the family hubs because they do offer a lot of those services”*

2.Testimonials good for aiding the description of what to expect from therapy-

*“I think maybe just knowing exactly what to expect”* but need to outline what the intervention is beforehand *“So if was done a good job of like the information sharing leading up to it. So this is what the session is going to look like”*

3.Defining the term speech and language therapy, about working together-

“*And I think that conversation about therapy being working together as opposed to me doing to you, I'm not here to kind of take over”, ”It's collaborative”*

4.Verbalising small gains is important, and unique to each child *“And individual”*

**Rating:** 5- Very acceptable

Future things for the researchers to note:

1. Childcare consideration *“I just suddenly thought, what about childcare?”,* not raised directly by parents interviewed, clarify other children can attend the session with the parent if childcare is an issue.
