# Supplementary material for: Enhancing the Prospective Acceptability of a Paediatric Speech and Language Therapy Intervention With Parents From Under‐Represented Communities
Source: Health Expect. 2026 Aug 30;29(5):e70855. doi: 10.1111/hex.70855 (PMC13527411; doi:10.1111/hex.70855)
Supplement: Supplementary file 8 [file HEX-29-e70855-s008.docx]

**Supplementary material 3**

**Schedule for interview one**

1.Pre-interview questions/welcome

Thank you for helping us.

Re-check consent.

*Have you heard of speech and language therapy before?* (SLT is….)

*Have you done anything like this before?*

*What are you hoping to achieve by taking part?*

2.Video

Briefly describe aims (6 interviews and steering group role).

Show video.

Opportunity for questions/clarification.

Show video again if requested.

3.Interview questions

1.How do you feel about the intervention? *(affective attitude)*

Prompts: why?

2.How much effort do you think it took for *video character* to take part in the intervention? (*burden)*

Prompts: why? What could have made it less effortful for them?

3.Do you think the intervention is a good fit for this individual family? (*ethicality)*

Prompts: why/why not? How could it be a better fit?

4.Do you think the intervention will work for this family? *(perceived effectiveness)*

Prompts: why/why/not? What could be done to make it work better?

5.Is it clear how the intervention will help *video character* to develop their speech and language? *(intervention coherence)*

Prompts: Why/why not? What could make this clearer?

6.How confident do you think the *video character* would feel about taking part in the intervention? *(self-efficacy)*

Prompts: Why? What could increase their confidence?

7.Do you think that *video character* would be able to prioritise taking part in the intervention? *(opportunity costs)*

Prompts: Why/why not? What could help them with this?

8.General acceptability (*ask to rate)*

1- Completely unacceptable
2- Unacceptable
3- No opinion
4- Acceptable
5- Completely acceptable

4.Summary/close

Summarise key points together. Bullet point on whiteboard.

Reiterate- steering group and other interviews.

Thank you for taking part.

Review session in approx. 4 weeks.
