# Supplementary material for: Enhancing the Prospective Acceptability of a Paediatric Speech and Language Therapy Intervention With Parents From Under‐Represented Communities
Source: Health Expect. 2026 Aug 30;29(5):e70855. doi: 10.1111/hex.70855 (PMC13527411; doi:10.1111/hex.70855)
Supplement: Supplementary file 9 [file HEX-29-e70855-s009.docx]

**Supplementary materials 1**

**GRIPP 2 short form**

| **Section and item** | **Where stated within the article** | **Further elaboration/additional information** |
| --- | --- | --- |
| **Aim:** Aim of PPI in the study | Aim (end of introduction section) | Enhance prospective acceptability of a new speech and language therapy intervention |
| **Methods:** Clear description of the methods used for PPI in the study | Materials and methods section | 1:1 interviews with parent partners, online meeting with the project steering group |
| **Study results:** Outcomes- reported results of PPI in the study, including positive and negative outcomes | Results section | Refinements made to the intervention (table 3) |
| **Discussion and conclusions:** Outcomes—Comment on the extent to which PPI influenced the study overall. Describe positive and negative effects | Discussion section | Positive- PPI central to the study including co-designing the methods, which facilitated the participation of parent partners.  Negative- prospective acceptability is different to retrospective acceptability, continued involvement is important. |
| **Reflections/critical perspective:** Comment critically on the study, reflecting on the things that went well and those that did not, so others can learn from this experience | Discussion section | Benefits to a flexible approach; Limitation- not having an in person interpreter, plan for this in funding applications. |
